# An intragenic duplication in the AFF2 gene associated with Cornelia de Lange syndrome phenotype

**DOI:** 10.3389/fgene.2024.1472543

**Published:** 2024-11-01

**Authors:** Cristina Lucia-Campos, Ilaria Parenti, Ana Latorre-Pellicer, Marta Gil-Salvador, Ilaria Bestetti, Palma Finelli, Lidia Larizza, María Arnedo, Ariadna Ayerza-Casas, Julia Del Rincón, Laura Trujillano, Beatriz Morte, Luis A. Pérez-Jurado, Pablo Lapunzina, Elsa Leitão, Jasmin Beygo, Christina Lich, Fabian Kilpert, Sabine Kaya, Christel Depienne, Frank J. Kaiser, Feliciano J. Ramos, Beatriz Puisac, Juan Pié

**Affiliations:** ^1^ Department of Pharmacology and Physiology, Unit of Clinical Genetics and Functional Genomics, School of Medicine, University of Zaragoza, CIBERER-GCV02 and IIS-Aragon, Zaragoza, Spain; ^2^ Institute of Human Genetics, University Hospital Essen, University of Duisburg-Essen, Essen, Germany; ^3^ SS Medical Genetics Laboratory, SC Clinical Pathology, Foundation IRCCS Ca’ Granda Ospedale Maggiore Policlinico, Milan, Italy; ^4^ Department of Medical Biotechnology and Translational Medicine, Università degli Studi di Milano, Milan, Italy; ^5^ Experimental Research Laboratory of Medical Cytogenetics and Molecular Genetics, IRCCS Istituto Auxologico Italiano, Milan, Italy; ^6^ Unit of Paediatric Cardiology, Service of Paediatrics, University Hospital “Miguel Servet”, Zaragoza, Spain; ^7^ Clinical and Molecular Genetics Area, Vall d’Hebron Hospital, Medicine Genetics Group, Vall d’Hebron Research Institute (VHIR), Barcelona, Spain; ^8^ Centre for Biomedical Network Research on Rare Diseases (CIBERER), Instituto de Salud Carlos III, Madrid, Spain; ^9^ Genetics Service, Hospital del Mar Research Institute (IMIM), Barcelona, Spain; ^10^ Genetics Unit, University Pompeu Fabra, Barcelona, Spain; ^11^ Institute of Medical and Molecular Genetics (INGEMM), University Hospital “La Paz”-IdiPAZ, Madrid, Spain; ^12^ ERN-ITHACA, University Hospital La Paz, Madrid, Spain; ^13^ Center for Rare Diseases (Essener Zentrum für Seltene Erkrankungen, EZSE), University Hospital Essen, Essen, Germany; ^14^ Department of Paediatrics, Unit of Clinical Genetics, Service of Paediatrics, University Hospital “Lozano Blesa”, School of Medicine, University of Zaragoza, CIBERER-GCV02 and IIS-Aragon, Zaragoza, Spain

**Keywords:** AFF2, CdLS, intragenic duplication, familial case, X-inactivation, Oxford Nanopore Technologies

## Abstract

Cornelia de Lange syndrome (CdLS, OMIM #122470, #300590, #300882, #610759, and #614701) is a rare congenital disorder that affects the development of multiple organs and is characterized by physical abnormalities and cognitive and behavioral disabilities. Its molecular basis is mainly based on alterations in genes encoding structural and regulatory proteins related to the cohesin complex. Moreover, other transcriptional regulatory factors have been linked to this syndrome. However, additional causative genes are still unknown, since many patients still lack a molecular diagnosis. Herein, we describe a case with multiple affected family members presenting with an intragenic duplication in the AFF2 gene. The direct tandem intragenic duplication of exons 10, 11 and 12 was detected through high-resolution array Comparative Genomic Hybridization and next-generation sequencing technologies. Confirming the X-linked inheritance pattern, the duplication was found in the patient, his mother and his maternal aunt affected (dizygotic twins). Targeted sequencing with Oxford Nanopore Technologies revealed an aberrant transcript which is predominantly expressed in the patient and his aunt. Along with these results, a significant reduction in AFF2 gene expression levels was detected in these two individuals. Clinically both subjects exhibit a classic CdLS phenotype, whereas the mother is mostly unaffected. Consistent with the phenotypical differences observed between the mother and the aunt, there is a marked difference in X-inactivation patterns skewing. Given the crucial role of AFF2 in transcriptional regulation, it is not surprising that AFF2 variants can give rise to CdLS phenotypes. Therefore, the AFF2 gene should be considered for the molecular diagnosis of this syndrome.

## 1 Introduction

Regulation of gene expression is an essential process in cell biology, which occurs precisely and coordinately from the early developmental stages (Asami et al., 2022). Recently, the term chromatinopathies has been introduced to describe disorders affecting transcription and chromatin remodeling. These disorders are often accompanied by developmental delay, facial dysmorphism, and intellectual disability (Avagliano et al., 2020; Parenti and Kaiser, 2021).

One of the best characterized chromatinopathies is Cornelia de Lange syndrome (CdLS, OMIM #122470, #300590, #300882, #610759, #614701), a congenital disorder affecting the development of multiple organs. Its prevalence is estimated to be about 1/10,000-30,000 live births (Kline et al., 2007). Clinically, it is characterized by distinctive facial features, prenatal and postnatal growth retardation, as well as cognitive impairment and behavioral disturbances (Ajmone et al., 2014; Pié et al., 2016; Grados et al., 2017; Kline et al., 2018). However, not all individuals with the syndrome exhibit the typical phenotype. The clinical features of the disorder can vary widely, ranging from mild to severe, and even overlap with the phenotypes of other similar syndromes, such as Rubinstein-Taybi, KBG, Coffin-Siris, or Wiedemann-Steiner (Parenti et al., 2017; Cucco et al., 2020).

To date, CdLS has been associated with several types of genetic variations, including missense, nonsense, splicing, and copy number variations (CNVs) (Teresa-Rodrigo et al., 2014; Kline et al., 2018). These variants affect eight main genes: *NIPBL*, *SMC1A*, *SMC3*, *RAD21*, *HDAC8*, *BRD4*, *MAU2,* and *ANKRD11* (Gil-Rodríguez et al., 2015; Huisman et al., 2017; Kline et al., 2018; Krab et al., 2020; Parenti et al., 2020; Selicorni et al., 2021). Variants in the *NIPBL* gene account for approximately 70% of cases, with more than 10% of them involving postzygotic mosaicism (Latorre-Pellicer et al., 2021). The proteins encoded by the aforementioned genes are all involved in transcriptional regulation as well as chromatin remodeling processes (Kline et al., 2018). In terms of molecular implications, there is increasing evidence linking CdLS to global alterations of gene expression (Yuan B et al., 2015; Izumi, 2016; Mills et al., 2018; Garcia et al., 2021). Recent studies in cortical neurons from CdLS patients have revealed transcriptional alterations in genes associated with neuronal functions, including synaptic transmission or signaling processes (Weiss et al., 2021).

Given the extensive phenotypic and genetic variability of the syndrome, consensus criteria have been established to aid the clinical diagnosis and to classify the condition into classical and non-classical forms, both falling within the CdLS spectrum (Kline et al., 2018). However, 15% of patients with a CdLS-like phenotype still remain without a clear genetic diagnosis.

In this study, we report an intragenic duplication within the *AFF2* gene in three individuals of the same family. CCG triplet repeats of the 5′ untranslated region (UTR) of this gene have been described as causative for Fragile XE syndrome (FRAXE, OMIM #309548) (Gu et al., 1996; Hillman and Gecz, 2001). Additionally, intragenic deletions have also been described (Gecz et al., 1996; Stettner et al., 2011). However, the full spectrum of clinical features associated with variants in *AFF2* still needs to be assessed.

In this work, we analyzed the consequences of the *AFF2* duplication through the study of gene expression in three family members. We subsequently compare the clinical features of the reported individuals with those of FRAXE syndrome, *AFF2* variants and CdLS. Our data suggest that *AFF2* may be considered a novel causal gene within the broad CdLS spectrum.

## 2 Materials and method

### 2.1 Clinical diagnosis

Clinical data have been collected at the University Hospital “Lozano Blesa” using a standard restricted-term questionnaire. Detailed phenotypes of the individuals have been evaluated by the patient’ clinician using the Human Phenotype Ontology (HPO) nomenclature for better comparison. The severity was determined according to the clinical score published by Kline and colleagues in 2018 (Kline et al., 2018). Additionally, as a complementary tool, Face2Gene technology was used to analyze the facial photographs of the individuals in the study (Gurovich Y et al., 2019; Latorre-Pellicer et al., 2020).

The biological samples have been collected from the sample collection C.0002316, registered in the Institute of Health Carlos III (ISCIII) for the investigation on the Cornelia de Lange syndrome, whose principal investigator is Prof. Feliciano J. Ramos.

The study has been performed according to the Declaration of Helsinki protocols and approved by the Regional Ethics Committee of Clinical Research from the Government of Aragón (CEICA; PI15/00707, PI19/01860). Informed consent was obtained from all subjects included in this study. Additional permission and informed consent were obtained for the publication of the photographs.

### 2.2 Analysis of the *AFF2-*related phenotypic spectrum

This analysis was performed by reviewing all the relevant clinical information about *AFF2* variants reported in the literature and databases, including missense, nonsense, small deletions, small insertions variants and intragenic deletions and duplications, as well as all associated phenotypes. All cases were retriev from PubMed (https://pubmed.ncbi.nlm.nih.gov/), ClinVar (https://www.ncbi.nlm.nih.gov/clinvar/), and DECIPHER (https://www.deciphergenomics.org/). We only took into consideration variants associated with clinical descriptions.

### 2.3 Molecular diagnosis

#### 2.3.1 Cell culture

Human primary fibroblasts were obtained from a skin biopsy from the anterior thigh. Fibroblasts were cultured in Dulbecco’s Modified Eagle Medium (DMEM, HyClone™) supplemented with 10% fetal bovine serum (FBS, Gibco™), 1% streptomycin/penicillin (HyClone™), under the conditions of a humidified atmosphere at 37°C with 5% CO_2_ in the incubator. The same protocol was used for all the cell lines. Cells were cultured between passages 1 and 4. Before isolating the total DNA and RNA, the cell lines were tested to verify the absence of *Mycoplasma* contamination.

#### 2.3.2 Isolation of DNA and RNA

Genomic DNA and RNA isolation were performed from blood cells and fibroblast samples. Lymphocytes derived’ DNA was extracted using a conventional phenol–chloroform isoamyl alcohol method. The DNA from fibroblast was isolated using the PureLink™ Genomic DNA kit (Invitrogen, Thermo Fisher Scientific), according to the manufacturer’s protocol. Isolation of RNA was performed from both blood and fibroblast lines using the Total RNA Purification kit (Norgen Biotech) according to the manufacturer’s protocol. The purity and integrity of the nucleic acids were assessed by electrophoresis and spectrophotometry using NanoDrop ND-2000 spectrophotometer (Thermo Fisher Scientific).

#### 2.3.3 DNA analyses

##### 2.3.3.1 Exome sequencing and reanalysis

Whole-exome sequencing was performed using blood derived’ genomic DNA of the patient (II-1) in a private laboratory (qGenomics S.L.). Then, the clinical and genomic raw data from the patient (II-1) were submitted to the Undiagnosed Rare Diseases Programme (EnoD; https://www.ciberer.es/en/transversal-programmes/scientific-projects/undiagnosed-rare-diseases- programme-enod) for more in-depth investigation. This programme is an initiative of the Center for Biomedical Network Research on Rare Diseases (CIBERER) to identify the genetic cause of rare diseases in selected undiagnosed cases (Luque et al., 2022). Results are periodically reassessed by an expert team using the software associated with the EnoD Programme (Bullich et al., 2022).

##### 2.3.3.2 Array Comparative Genomic Hybridization

Human Genome Comparative Genomic Hybridization (CGH) Microarray kit 1 × 400 K was performed on genomic DNA from blood of the patient (II-1) according to the manufacturer’s instructions (Agilent Technologies). Data were extracted and analyzed for copy number changes using Agilent CytoGenomics v.3.0 software (Agilent Technologies). Copy number variations (CNVs) were analyzed and mapped using the Human Genome assembly GRCh37/hg19. CNV classification was performed according to the Database of Genomic Variants (DGV) (http://projects.tcag.ca/variation/) to exclude common polymorphic CNVs with a frequency >1% in healthy population.

##### 2.3.3.3 Deep-sequencing Cornelia de Lange syndrome gene panel

The fibroblast´s genomic DNA from the patient (II-1) and blood´s genomic DNA from the mother (I- 2) and the maternal aunt (I-3) were sequenced using a deep-sequencing panel including 35 genes related with CdLS, with a total target region of 249.25 kb. Library preparation, emulsion PCR, bead enrichment, and chip loading were automatically performed on an Ion Chef™ instrument (Thermo Fisher Scientific) using Ion AmpliSeq™ Kit for Chef DL8 and 530™ Kits (Thermo Fisher Scientific) according to the manufacture’s protocols. Templates were sequenced on an Ion S5™ XL sequencer (Thermo Fisher Scientific) using 530™ Kits with a read length of 200. Raw data analysis was carried out with Torrent Suite SoftwareTM (v.5.12.3). After alignment to the hg19 human reference genome, the annotation of SNVs, indels and CNVs was performed using the Ion Reporter SoftwareTM (v.5.20.2.0). The sequencing data were evaluated using Integrative Genomics Viewer (IGV) (http://software.broadinstitute.org/software/igv/).

##### 2.3.3.4 Real-time quantitative PCR (qPCR)

Confirmation of the coding regions involved in the CNV was performed on genomic DNA from blood from all available family members (II-1, I-1, I-2, I-3, I-4, I-5). The analysis was screened by real-time quantitative PCR (qPCR) using an NZYSupreme qPCR Green Master Mix (2x) ROX (NZYtech) on QuantStudio™ 5 System (Applied Biosystems). Samples were assessed in triplicate in each experiment and two biological replicates were performed. Data were analysed through the 2^−ΔΔCT^ method (Livak and Schmittgen, 2001). Statistical analyses and graphics were generated with GraphPad Prism 9 software. The primer pairs bind to inner regions of exons in the gene of interest, and their sequences and PCR conditions are available upon request.

#### 2.3.4 RNA analyses

##### 2.3.4.1 cDNA synthesis and analysis

Total RNA isolated from blood and fibroblast lines was retrotranscribed into single-strand cDNA using a SuperScript™ First-Strand Synthesis System for RT-PCR kit (Invitrogen, Thermo Fisher Scientific) following the manufacturer’s protocol. cDNA synthesis was performed using 2 µg of total RNA and random hexamers as primers.

Sanger sequencing was performed to localize the duplication breakpoints. This analysis was carried out on cDNA from blood and fibroblasts from the patient (II-1), as well as from his father (I-1), his mother (I-2) and his maternal aunt (I-3). PCRs were performed using a KAPA2G Robust PCR kit (KAPA BIOSYSTEMS). Primer sequences and PCR conditions are available upon request. Amplicons were sequenced using a BigDye™ Terminator v.3.1 Cycle Sequencing kit (Thermo Fisher Scientific). Deletion junction sequences were aligned to the human reference genome sequence (GRCh38/hg38), and electropherograms were analyzed with the ChromasPro 1.5 software (Technelysium Pty Ltd.).

##### 2.3.4.2 Targeted sequencing with Oxford Nanopore Technologies (ONT)

RNA from fibroblasts was retrotranscribed with the LunaScript^®^ RT SuperMix Kit (NEB, Basdorf, Germany), following manufacturer’ instruction. The full length *AFF2* transcript (ENST00000370460.7, NM_002025.4) was amplified from cDNA of the patient (II-1), his mother (I-2) and his maternal aunt (I-3) by touch-down PCR using PrimeSTAR GXL DNA Polymerase (Takara Bio) with 10% DMSO (Primer sequences are available upon request). The PCR products were purified using the DNA Clean & Concentrator (Zymo Research). A library of multiplexed samples was prepared using the SQK-LSK109 ligation-based sequencing kit (Oxford Nanopore) and the native barcoding protocol (EXP-NBD196). In summary, 200 fmol of each purified amplicon were incubated with NEBNext Ultra II End Repair/dA-tailing Module Reagents (NEB) at 20°C for 5 min and 65°C for 5 min, after which, barcodes were ligated using the NEB Blunt/TA Ligase Master Mix at 20°C for 20 min and 65°C for 10 min. Barcoded amplicons were pooled and purified using AMPure XP beads (Beckman Coulter), adapters were ligated incubating during 10 min with Adapter Mix II Expansion/NEBNext Quick Ligation Reaction Module followed by clean-up with AMPure XP beads. Approximately 15 ng of the library were loaded onto a MinION R9.4.1 FLO-MIN106 flow cell sequencing for up to 24 h while monitoring using the MinKNOW software. All steps were performed following the manufacturer’s protocols. Guppy (version 6.5.7) (Community N. Downloads - Release notes. Oxford Nanopore Technologies) was used for base-calling, pycoQC (version 2.5.2) (Leger and Leonardi, 2019) and NanoPlot (version 1.41.6) (De Coster and Rademakers, 2023) for quality control. The command line version of Guppy, guppy_basecaller, used the “sup” model and the parameters: “--recursive -- compress_fastq --do_read_splitting --calib_detect--records_per_fastq 0 -- enable_trim_barcodes.” Reads were aligned with minimap2 (version 2.28-r1209) (Li, 2018) to a reference consisting of NM_002025.4 concatenated exons. Variants were called with Sniffles2 (version 2.2) (Smolka et al., 2024) as well as the reference number and variant reads.

##### 2.3.4.3 Gene expression analysis

Global expression levels of *AFF2* (NM_002025.4) in fibroblasts were assessed by qPCR using NZYSupreme qPCR Green Master Mix (2x) ROX (NZYtech) on QuantStudio™ 5 System (Applied Biosystems). Six healthy controls (three male and three female) were used for comparison. 1 μL of cDNA and 2 µM of primers were used in a volume of 10 µL for each reaction. Samples were assessed in triplicate in each experiment and three biological replicates were performed. Primer sequences are available upon request. The global gene expression levels were calculated normalizing to the housekeeping *β-actin* gene and compared with the controls. The Ct values for each sample were determined with amplification plots in the logarithmic phase. The PCR outcome and efficiency of amplification were determined using QuantStudio™ Design and Analysis Software v1.5.1 using the 2^−ΔΔCT^ method. Statistical analyses and graphics were generated with GraphPad Prism 9 software.

#### 2.3.5 Assessment of X chromosome inactivation

The level of skewing of X chromosome inactivation (XCI) was evaluated by studying the methylation status at two polymorphic regions of the X chromosome. Specifically, the highly polymorphic region (CAG repeats) in exon 1 of the androgen receptor (*AR*) gene was explored. Digestions with methylation-sensitive restriction enzymes HpaII and HhaI were carried out at both blood and fibroblas genomic DNA from the mother (I-2) and maternal aunt affected (I-3). In addition, the highly polymorphic region (CGG repeats) of the *FMR1* gene was also evaluated. Digestion with the methylation-sensitive restriction enzyme HpaII was carried out at both blood and fibroblast genomic DNA from the mother (I-2) and the maternal aunt (I-3). For both loci, PCR amplification of digested and undigested DNA was performed. Finally, the allele ratios were calculated (Allen et al., 1992; Carrel and Willard, 1999). We considered alleles separated by more than two trinucleotide repeats to be informative. For skewed XCI, we used a cutoff of >80:20%, and for extremely skewed XCI, a cutoff 241 of >95:5%.

## 3 Results

### 3.1 Clinical report

#### 3.1.1 Patient (II-1)

**FIGURE 1:**
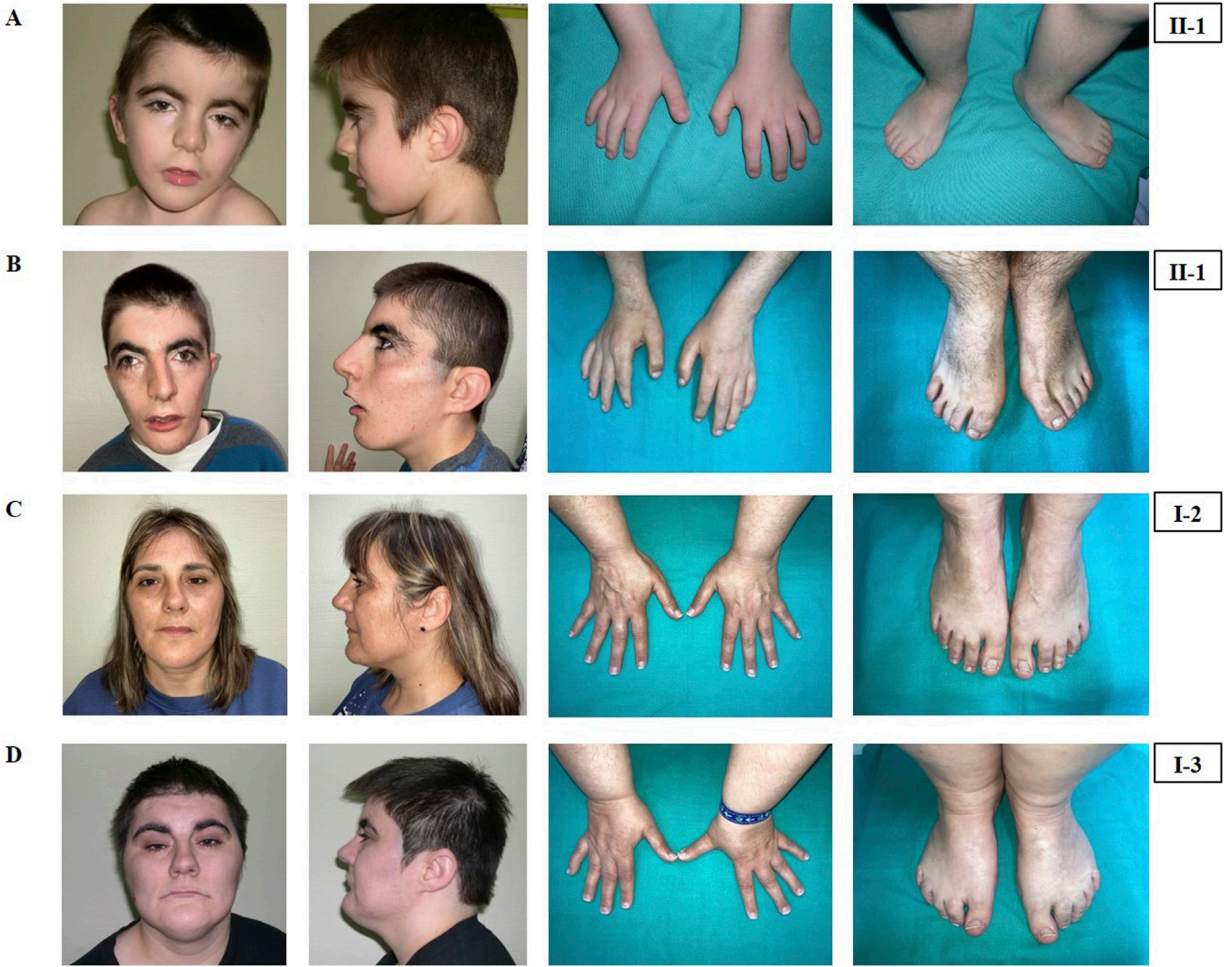
Phenotypical characteristics of the patient (II-1), his mother (I-2) and his maternal aunt affected (I-3). Craniofacial features, hands and feet of the patient (II-1) at the age of 4 years **(A)** and at the age of 19 years **(B)**. Craniofacial features, hands and feet of the mother (I-2) **(C)** and of the maternal aunt (I-3) **(D)**.

The index patient of this study is an adolescent male (II-1, see Figures 1A, B) born from a non-consanguineous couple at 35 weeks of gestational age by Cesarean section. His birth weight was 1.640 kg (−2.29 SD), length 44 cm (−1.1 SD) and head circumference 29.5 cm (−1.36 SD). Due to the low birth weight and the presence of a congenital atrial septal defect, he was admitted to the Neonatal Intensive Care Unit (NICU).

He was clinically diagnosed with CdLS at the age of 4 years (Figure 1A). The following features supported CdLS diagnosis: synophrys (HP:0000664), thick eyebrows (HP:0000574), long philtrum (HP:0000343), thin upper lip vermilion (HP:0000219) with downturned corners of the mouth (HP:0002714), microcephaly (HP:0000252), and short fifth finger (HP:0009237) (Figures 1A, B). He additionally presented with cognitive impairments, abnormal heart morphology (HP:0001627), absent speech (HP:0002300), hyperactivity (HP:0000752), and a short attention span (HP:0000736). Psychiatric assessment was carried out at the age of 13 years using the Brunet-Lezine BL-EC Scale and suggested a developmental quotient of around 24 months. A summary of the clinical features of the patient (II-1) is shown in Table 1.

**TABLE 1:** Clinical features of the patient (II-1), his mother (I-2) and his maternal aunt affected (I-3) compared with the clinical features of FRAXE syndrome, copy number variants affecting the *AFF2* gene and Cornelia de Lange syndrome.

Clinical features	FRAXE syndrome	Variants *AFF2*	CdL syndrome	Patient (II-1)	Mother (I-2)	Maternal aunt (I-3)
Global developmental delay HP:0001263						
Growth delay HP:0001510						
Prenatal growth retardation HP:0001511						
Postnatal growth retardation HP:0008897						
Abnormal skull morphology HP:0000929						
Microcephaly HP:0000252						
Brachycephaly HP:0000248						
Dysmorphic facial features HP:0001999						
Synophrys HP:0000664						
Arched, thick eyebrows HP:0002553, HP:0000574						
Long eyelashes HP:0000527						
Proptosis HP:0000520						
Palpebral ptosis HP:0000508						
Ocular hypertelorism HP:0000316						
Upturned nasal tip HP:0000463						
Long, smooth philtrum HP:0000343, HP:0000319						
Thin upper lip vermilion HP:0000219						
Downturned corners of the mouth HP:0002714						
High arched palate HP:0000218						
Dental crowding HP:0000678						
Low anterior hairline HP:0000294						
Narrow face HP:0000275						
Abnormality of limbs HP:0040064 and musculoskeletal system HP:0033127						
Small hands HP:0200055						
Clinodactyly HP:0004209						
Short fifth finger HP:0009237						
Small feet HP:0001773						
Vertebral anomalies HP:0003468						
Broad chest HP:0000914						
Neurodevelopmental abnormality HP:0012759						
Intellectual disability HP:0001249						
Specific learning disability HP:0001328						
Delayed speech and language development HP:0000750						
Abnormal social communication behavior HP:0034434						
Behavior disorders HP:0000708						
Attention déficit disorder HP:0007018						
Hyperactivity HP:0000752						
Aggressive behavior HP:0000718						
Autistic behavior HP:0000729						
Other phenotypic abnormality HP:0000118						
Seizures/Epilepsy HP:0001250						
Hearing impairment HP:0000365						
Cardiovascular anomalies HP:0002564						
Apnea HP:0002104						
Gastroesophageal reflux HP:0002020						
Vesicoureteral reflux HP:0000076						
Recurrent infections HP:0002719						
Hirsutism HP:0001007						

While blue cells indicate presence of the feature, beige cells indicate its absence.

The latest evaluation was performed at 19 years of age. At this time, his clinical features remained consistent with the initial CdLS diagnosis (Figure 1B). He additionally presented with gastroesophageal reflux (HP:0002020) and self-injurious behavior (HP:0100716).

**FIGURE 2:**
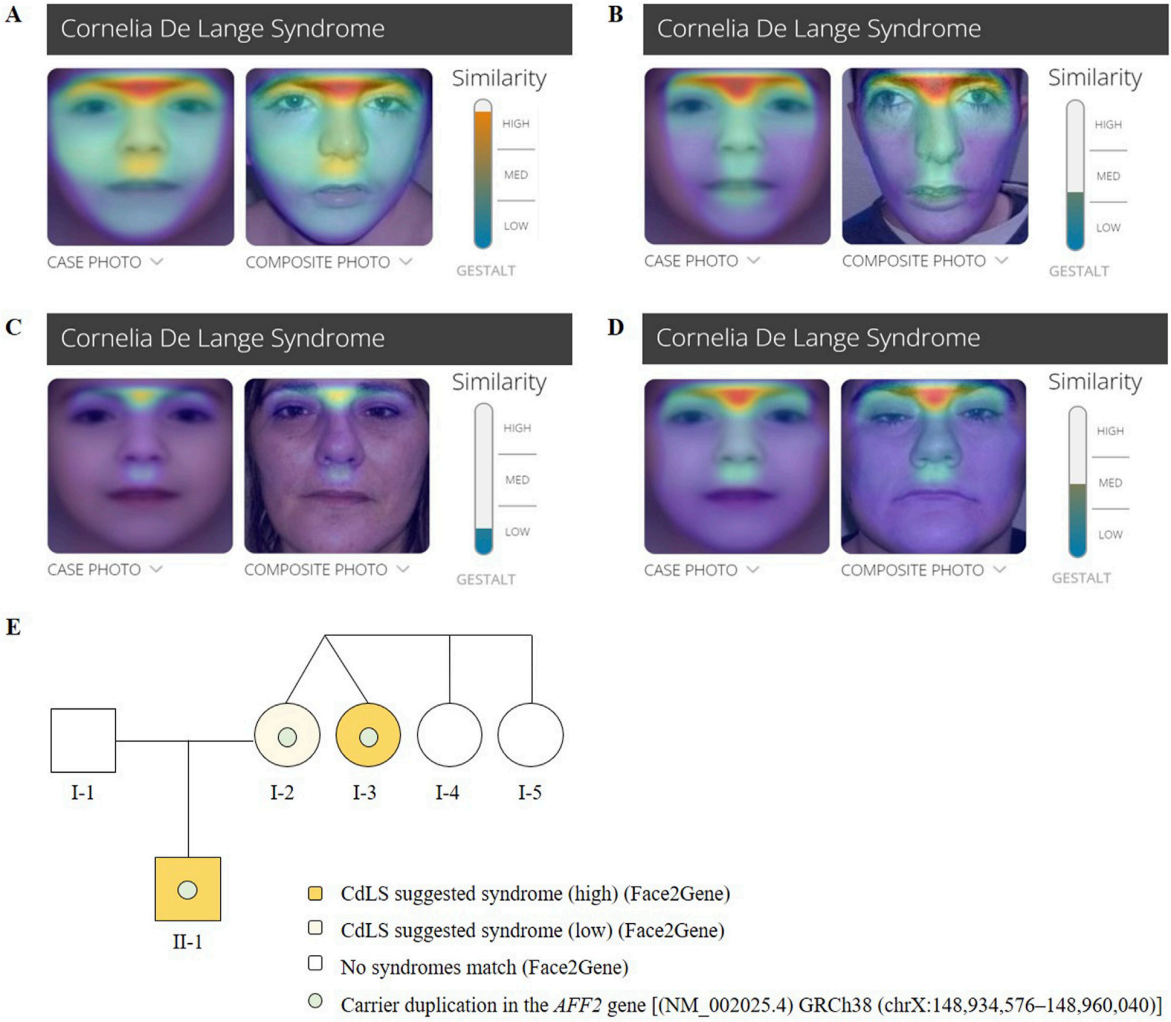
Face2Gene evaluation of facial photographs of the **(A)** patient (II-1) at the age of 4 years, **(B)** the patient (II-1) at the age of 19 years, **(C)** the mother (I-2) and **(D)** the maternal aunt affected (I- 750 3). **(E)** Pedigree chart of the family showing Mendelian segregation of the *AFF2* (NM_002025.4) 751 duplication [GRCh38 (chrX:148,934,576–148,960,040)], as well as the results of the Face2Gene phenotypic analysis.

The calculated clinical CdLS score was 13 (Supplementary Table S1). According to the consensus criteria, he would be included in the classic CdLS phenotype group. Further clinical analysis was conducted with Face2Gene at ages 4 (Figure 2A) and 19 year old (Figure 2B), and the prediction showed high and medium CdLS Gestalt similarity, respectively.

#### 3.1.2 Mother (I-2)

The mother of the index patient presented with some milder features such as brachycephaly (HP:0000248), long philtrum (HP:0000343), and thin upper lip vermilion (HP:0000219) (Figure 1C). The clinical evaluation yielded a CdLS score of 6 (Supplementary Table S1), which is below the threshold for a classification of non-classic CdLS. Face2Gene revealed that she exhibits a low similarity to the CdLS Gestalt features (Figure 2C).

#### 3.1.3 Maternal aunt (I-3)

The maternal aunt affected of the index patient (I-3, mother’s dizygotic twin) also exhibits a CdLS phenotype (Figure 1D). The clinical diagnosis of CdLS was primarily based on the following dysmorphic facial features: synoprhys (HP:0000664), thick eyebrows (HP:0000574), long and smooth philtrum (HP:0000343, HP:0000343), thin upper lip vermilion (HP:0000219), downturned corners of the mouth (HP:0002714), and microcephaly (HP:0000252) (Figure 1D). Moreover, she presented with moderate intellectual disability (HP:0002342), global developmental delay (HP:0001263), postnatal growth retardation (HP:0008897), small hands (HP:0200055), small feet (HP:0001773), and hirsutism (HP:0001007) (more detailed clinical features are shown in Table 1).

The clinical evaluation yielded a CdLS score of 14 (Supplementary Table S1), consistent with a diagnosis of classic CdLS, as per the consensus criteria. Face2Gene result indicates a medium similarity with CdLS Gestalt (Figure 2D). In contrast, the two other sisters (I-4 and I-5) did not display any signs compatible with the diagnosis of CdLS (Figure 2E).

### 3.2 Molecular diagnosis

#### 3.2.1 DNA molecular analyses

**FIGURE 3:**
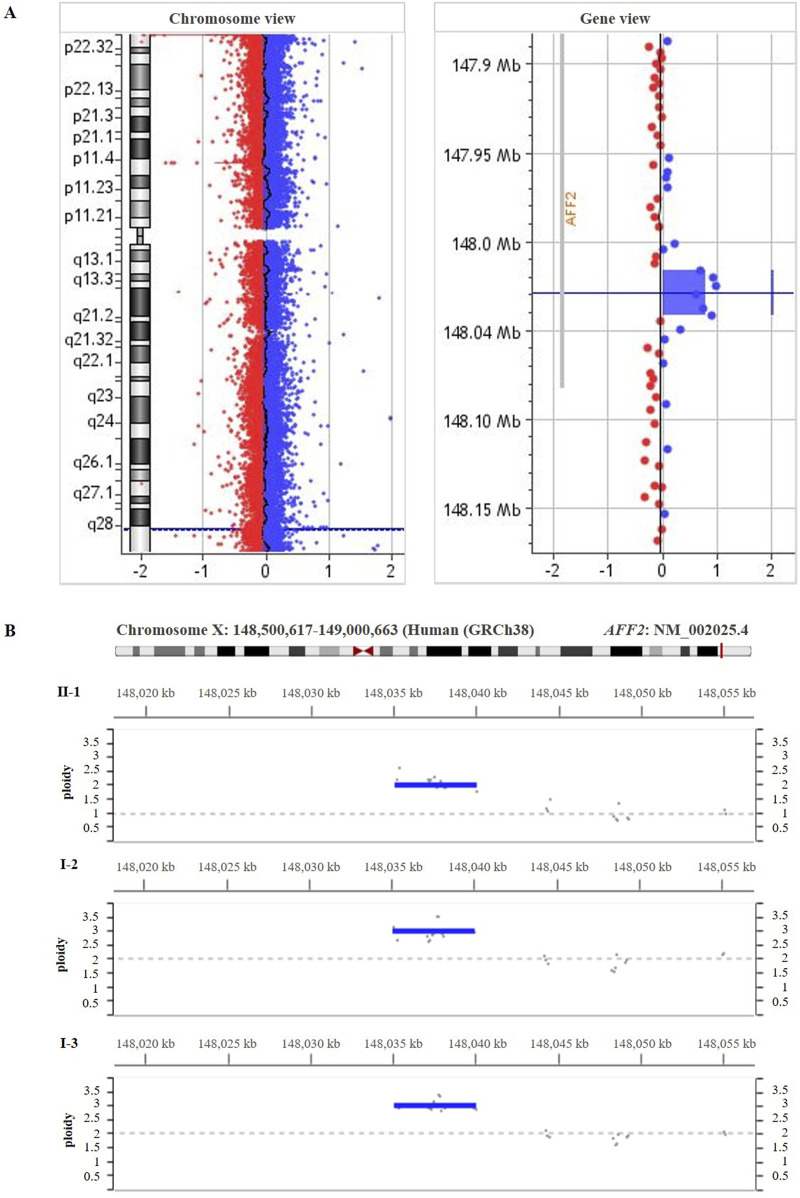
**(A)** High-resolution array-CGH 400 K performed on genomic DNA from the patient’s blood (II-1) showed a duplication spanning ~ 25.5 kb [arr(GRCh38) Xq28(148,934,576 – 148,960,040)x2)] involving part of the coding region of *AFF2* gene (NM_002025.4). **(B)** The deep-sequencing panel of gene amplicons specific for Cornelia de Lange syndrome result showed the duplication in *AFF2* [(NM_002025.4) GRCh38 (chrX:148,95,3536-148,958,504)] in the patient (II-1), his mother (I-2) and his maternal aunt affected (I-3).

285 Exome sequencing of the patient (II-1) did not reveal any pathogenic or likely-pathogenic single nucleotide variant. High-resolution array-CGH analysis of the patient (II-1) revealed an intragenic duplication in the *AFF2* gene (NM_002025.4). This duplication is described neither in population genomic databases nor in patients with neurodevelopmental disorders or our internal database of patients with CdLS. The chromosomal coordinates of the duplication are chrX:148,934,576- 148,960,040 (GRCh38/hg38) (Figure 3A), spanning from intron 9 to intron 12 and harboring exons 10, 11, and 12 of the *AFF2* gene. The reanalysis of exome data within the Program for Undiagnosed Rare Diseases (ENOD) detected the intragenic duplication in the *AFF2* gene (NM_002025.4). In addition, segregation analyses in the family were carried out through deep-sequencing (>1,000x) using CdLS gene panel (Figure 3B) and qPCR (Supplementary Figure S1), and both confirmed the intragenic duplication in the *AFF2* gene in the patient (II-1) in hemizygosity, as well as in the mother (I-2) and the maternal aunt (I-3) who are heterozygous carriers.

#### 3.2.2 Breakpoint identification

To investigate if the duplicated sequence was arranged in tandem, amplification of a cDNA fragment using a forward primer located distal to exon 12 and a reverse primer located proximal to exon 10 was performed. By doing this, a PCR product could be detected in the patient (II-1), his mother (I-2) and his maternal aunt (I-2), but not in the father (I-1) (Supplementary Figure S2A). Sanger sequencing of the aberrant product confirmed that the duplication is arranged in a direct tandem orientation (Supplementary Figure S2B), consisting of exons 10, 11, and 12 followed by a repeated identical sequence of exons 10, 11 and 12.

The analysis of the aberrant transcript sequence revealed the additional presence of the following missense substitution: p.(Glu897Asp) at the breakpoint. Notably, the aberrant transcript could potentially be translated into a mutant protein carrying 431 additional amino acids. This would include a duplication of the serine-rich domain, the two bipartite nuclear localization signals and the AF4 interaction motif (Supplementary Figure S3).

#### 3.2.3 Relative expression of the *AFF2* transcripts

**FIGURE 4:**
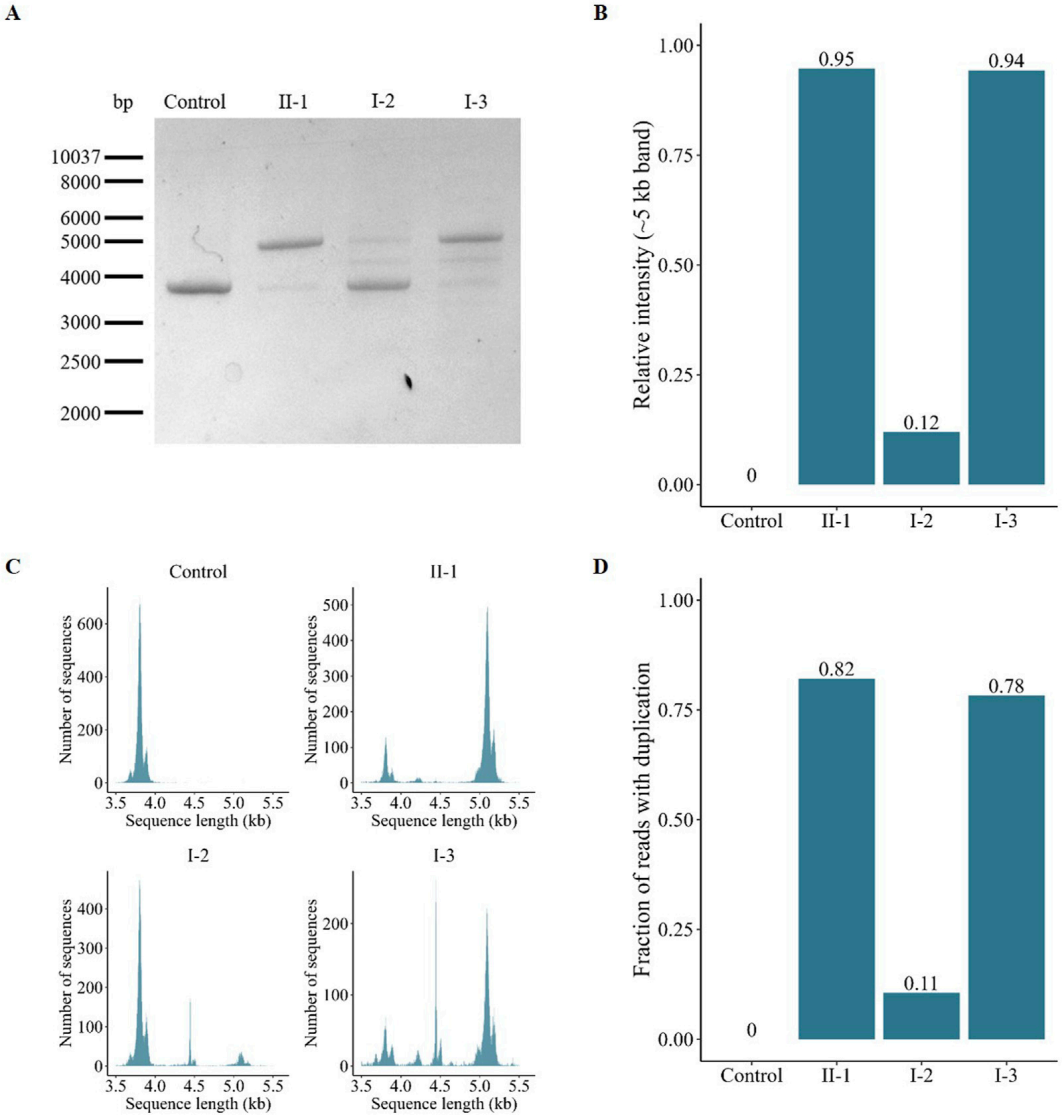
Characterization and quantification of the *AFF2* aberrant transcript. **(A)** Gel electrophoresis of the amplified full-length *AFF2* transcript. The control sample exclusively expresses the wild-type allele (≈4 kb). In contrast, the patient (II-1), his mother (I-2) and his maternal aunt affected (I-3) express both the wild-type and the duplicated allele (≈5 kb) at varying ratios. **(B)** Relative intensity of the PCR bands of the duplicated allele. The patient (II-1) and his maternal aunt (I-3) predominantly express the duplicated allele, whereas the unaffected mother (I-2) mainly expresses the wild-type allele. **(C)** Size-based distribution of the number of reads obtained after targeted sequencing with Oxford Nanopore Technologies. The peak around 4 kb represents the wild-type allele, while the peak around 5 kb corresponds to the mutant allele with the duplication of exons 10, 11, and 12. The middle peak around 4.5 kb, observed primarily on the mother (I-2) and the maternal aunt (I-3), is an unspecific product that maps at the centrosome region of chromosome 16. **(D)** Fraction of reads with the duplication obtained after targeted sequencing with Oxford Nanopore Technologies.

The full-length *AFF2* transcript was amplified by PCR. A single band of 3,933 bp corresponding to the wild-type *AFF2* construct was evident in the healthy control (Figure 4A). Individuals II-1, I-2, and I-3, however, showed an additional band, indicating the presence of an aberrant transcript (1,293 bp larger, with exons 10, 11 and 12 duplicated). The ratios between these two transcripts varied among the analyzed individuals. Specifically, the patient (II-1) and his maternal aunt affected (I-3) predominantly express the aberrant transcript, with relative expression levels calculated at 95% and 94% of the total, respectively, based on band intensities (Figure 4B). In contrast, the unaffected mother (I-2) primarily expresses the wild-type allele, with the aberrant allele accounting for only 12% of the total (Figure 4B).

Furthermore, an additional intermediate band was detected in the samples from both the mother (I-2) and the maternal aunt (I-3). To elucidate its origin, we utilized Oxford Nanopore Technology for comprehensive analysis of the amplified full-length transcripts. This approach confirmed the presence of the wild-type transcript (Figure 4C, peak around 4 kb) and confirmed the exons composition of the aberrant transcript (peak around 5 kb). However, reads corresponding to the intermediate amplicon (peak around 4.5 kb) mapped to the centromeric region of chromosome 16, indicating that this amplicon is rather a nonspecific PCR artifact. Analysis of relative read numbers corroborated the previous findings from the PCR band intensity analysis, showing that the patient (II-1) and his maternal aunt (I-3) predominantly express the aberrant transcript (82% and 78%, respectively), while the unaffected mother (I-2) primarily expresses the wild-type allele (89%) (Figure 4D).

#### 3.2.4 Total *AFF2* expression

**FIGURE 5:**
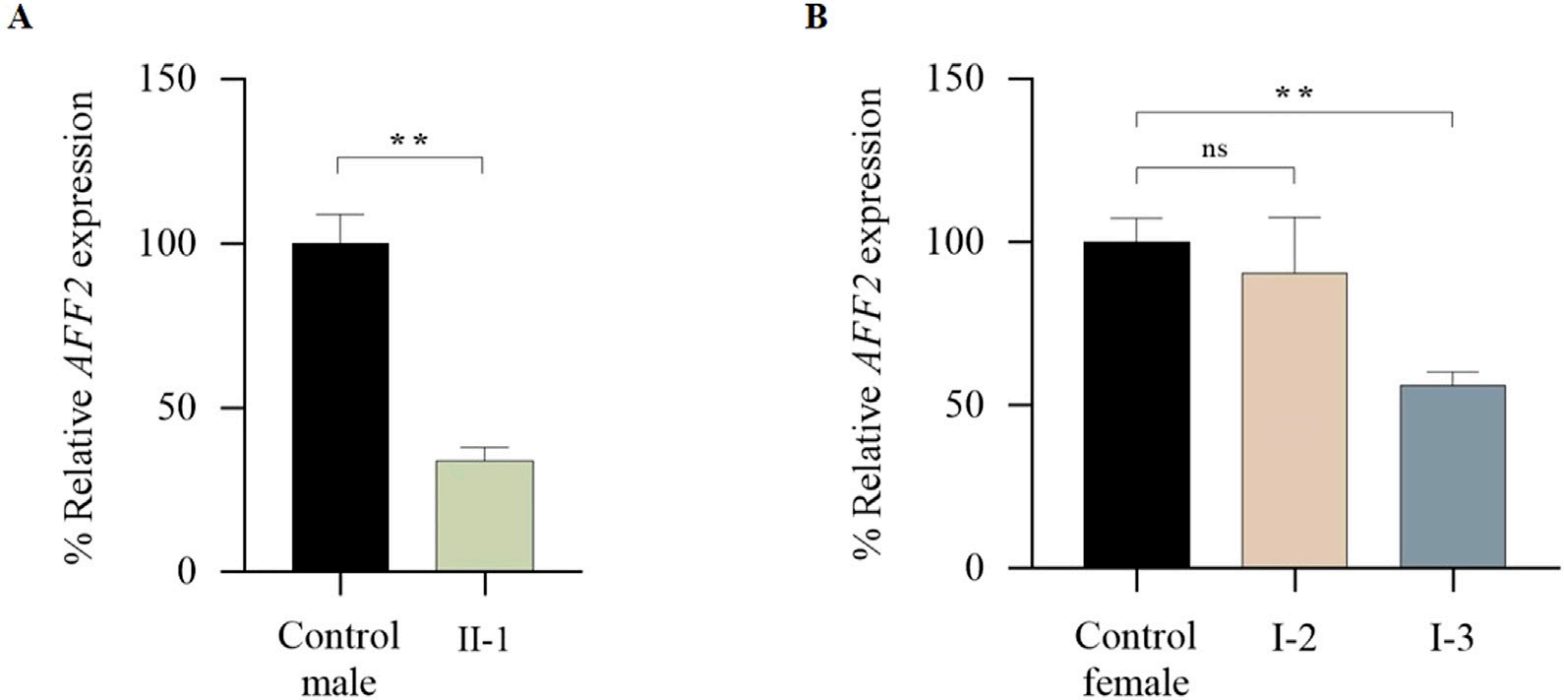
qPCR analysis of global expression levels of the *AFF2* gene (NM_002025.4). **(A)** Global expression levels of *AFF2* in the patient (II-1). Expression levels of *AFF2* (NM_002025.4) were normalized to actin. The expression level in male controls (n = 3) was arbitrarily set at 100%. **(B)** Global expression levels of *AFF2* in the mother (I-2) and the maternal aunt affected (I-3). Expression levels of *AFF2* (NM_002025.4) were normalized to actin. The expression level in female controls (n = 3) was arbitrarily set at 100%.

The total *AFF2* mRNA levels were assessed by qPCR. Expression levels of the patient (II-1) were normalized on the average expression levels of sex-matched healthy controls. The mean *AFF2* expression level in each individual was calculated as a percentage relative to the controls. While no significant changes were observed in *AFF2* mRNA levels in the mother (I-2 = 90,60%), a significant decrease of approximately 45% in total *AFF2* mRNA levels was noticed in the maternal aunt (I-3 = 56,16%), and a significant decrease of approximately 70% in total *AFF2* mRNA levels was observed in the patient (II-1 = 33.80%) (Figure 5).

#### 3.2.5 X-inactivation

The X-inactivation analysis was non-informative at the *AR* locus due to homozygosity. Analysis of the *FMR1* locus in the mother (I-2) revealed borderline skewing in favor of the wild-type allele (Allele 2) in blood (81/19) and random X-inactivation in fibroblast (36/64) (Table 2). On the other hand, the maternal aunt (I-3) similarly preferentially expresses the wild-type allele (Allele 2) in blood (83/17), but exclusively expresses the mutant allele (Allele 1) in fibroblasts (100/0) (Table 2).

**TABLE 2:** X-inactivation analysis.

Individual	DNA source	Locus	Allele 1:Allele 2
Mother (I-2)	Blood	*AR*	NI
I-2	Fibroblast	*AR*	NI
I-2	Blood	*FMR1*	81:19
I-2	Fibroblast	*FMR1*	36:64
Maternal aunt (I-3)	Blood	*AR*	NI
I-3	Fibroblast	*AR*	NI
I-3	Blood	*FMR1*	83:17
I-3	Fibroblast	*FMR1*	0:100

Individual studied, DNA, source, locus and the ratio of X-inactivation for each allele are specified. NI, indicates not informative (skewing.

could not be determined due to the homozygosity of the tested loci).

### 3.3 Systematic review of *AFF2* variants and their clinical features

A total of 50 *AFF2* variants were reviewed from ClinVar, DECIPHER and Pubmed and included in Supplementary Table S2. Among them, of which 7 were reported in case reports, 27 were published in studies of large cohorts of families or patients with intellectual disability, autism or epilepsy, 11 were reported in ClinVar as likely pathogenic or pathogenic and five were reported in DECIPHER. The type of the mentioned variants were intragenic duplications (6 cases), intragenic deletions (12 cases), small deletions (1 case), small insertions (3 cases) and SNVs such as nonsense (4 cases), missense (21 cases), and splicing (3 cases) variants (Supplementary Table S2).

Although there is no detailed clinical description of these cases, most are associated with intellectual disability (HP:0001249) (23/50) or autism (HP:0000717) (30/50). In cases in which data on facial dysmorphism were available, some of the traits that the individuals manifest were epicanthal folds (HP:0000286) (Gedeon et al., 1995), divergent strabismus (HP:0020049) (Gedeon et al., 1995), synophrys (HP:0000664) (Valentino et al., 2021), prominent nasal bridge (HP:0000426) (Gedeon et al., 1995; Sahoo et al., 2011), anteverted nares (HP:0000463) (Valentino et al., 2021), cleft palate (HP:0000175) (DECIPHER ID 273134, ClinVar VCV000986210.2) or widely spaced teeth (HP:0000687) (Gedeon et al., 1995) (Supplementary Table S2).

## 4 Discussion

The genetic etiology of Cornelia de Lange syndrome (CdLS) is mainly attributed to variants affecting the cohesin complex, and more specifically to heterozygous variants in the *NIPBL* gene (Kline et al., 2018; Alonso-Gil and Losada, 2023). However, an increasing number of patients with pathogenic variants in non-cohesin related genes with phenotypes that clinically overlap with CdLS are being described (Parenti et al., 2017; Avagliano et al., 2020; Eigenhuis et al., 2022; Shangguan and Chen, 2022). Thus, the term CdLS spectrum has emerged to include both classical and non-classical phenotypes of the syndrome (Kline et al., 2018).

In this study, we report a familial case with multiple affected family members presenting with a previously unreported intragenic duplication in the *AFF2* gene. After the first molecular evaluation, the causative CdLS genes known at that time were analyzed by Sanger sequencing but no pathogenic variation was revealed. The high-resolution array-CGH and the reanalysis of exome detected the intragenic duplication in the *AFF2* gene that could explain the patient’s phenotype. Familial segregation studies, performed by deep sequencing and qPCR supported the possible pathogenicity of the variant. The patient (II-1), his mother (I-2) and his maternal aunt affected (I-3) carried the duplication, while none of the maternal aunts unaffected harbored it. It was not possible to conduct the study in the maternal grandparents. In this case, the use of stepwise genetic diagnostic techniques enabled us to definitively confirm the identified intragenic duplication in the *AFF2* gene. This is an example of the difficulty of reaching a molecular diagnosis in patients with rare genetic diseases.

Studies at the mRNA level revealed a direct tandem duplication of exons 10, 11 and 12, which is common in these types of variants (Newman et al., 2015; Richardson et al., 2019). Implementing long-read sequencing using Oxford Nanopore Technologies allowed us to sequence and quantify the aberrant transcripts (Glinos et al., 2022). Through this technique, we detected an aberrant transcript that is more expressed in the patient (II-1) and his maternal aunt (I-3) in comparison to the mother (I-2), who exhibits minimal expression of the aberrant transcript. Conversely, global expression of the *AFF2* gene showed a significant decrease in total transcript levels in the patient (II-1) and his maternal aunt (I-3). This overall decrease in expression may result from mRNA quality control mechanisms that degrade aberrant transcripts (Łabno et al., 2016; Peck et al., 2019). The mother (I-2), who is largely unaffected, does not showed a reduction of the global expression. Thus, the preferential expression of the aberrant transcript, coupled with the decrease in global expression, accounts for the more severe phenotype of the patient (II-1) and his maternal aunt (I-3). This phenomenon has been described in other reported cases of intragenic duplications (Takahashi S et al., 2015; Zepeda-Mendoza et al., 2019).

Surprisingly, the patient (II-1) expresses 10%–20% of the wild-type transcript, which is not expected given his hemizygous status for the duplication. This observation suggests the presence of a mechanism that preserves wild-type mRNA during alternative splicing (Cetin et al., 2016). One possible underlying mechanism could be DNA methylation, which may influence alternative splicing and facilitate the rescue of the normal size transcript (Lev et al., 2015). On the other hand, the functional impact at the protein level in this case is challenging to predict. Intragenic duplications frequently result in a loss of function (Bertini et al., 2023). The additional 431 amino acids may alter the AFF2 structure due to the gain of functional domains and as a consequence affect its RNA-binding function (Bensaid et al., 2009).

Clinically, our index patient was classified as classic CdLS during his first evaluation at the age of four, with the diagnosis confirmed at his most recent evaluation. Furthermore, the application of artificial intelligence databases such as Face2Gene (Gurovich Y et al., 2019) further supports the CdLS diagnosis. Notably, an age-related evolution of his dysmorphic features was observed, involving particularly changes in the shape of his nose and the pronounced appearance of his eyes. Similar phenotypic changes with age have been described in other patients with CdLS (Latorre-Pellicer et al., 2021b; Jouret G et al., 2022).

Significant clinical differences are observed between his mother and his maternal aunt affected who are dizygotic twins. The maternal aunt (I-3), with a clinical CdLS score of 14, presents with classic phenotype. On the contrary, the mother (I-2) does not fall within the CdLS spectrum, even though some mild isolated features were observed. The difference in severity could be influenced by the variable level of skewing of the X-chromosome inactivation, as documented in other X-linked diseases (Sun et al., 2022). Although skewed X-chromosome inactivation was observed in both the mother (I- 2) and the maternal aunt (I-3) in blood, different inactivation rates were observed in fibroblasts. In the mother (I-2), X-chromosome inactivation was found to be random in fibroblasts, whereas in the maternal aunt (I-3) it was found to be fully skewed towards the silenced allele in blood. Furthermore, decreased AFF2 expression was observed in the maternal aunt (I-3) but not in the mother (I-2). Therefore, the skewed X-chromosome inactivation and the differential expression between the twins could potentially explain their distinct phenotypes.

The literature review showed that variants affecting the *AFF2* gene are mainly associated with the Intellectual Developmental Disorder X-Linked 109 (FRAXE, OMIM, #309548), a rare type of intellectual disability without well-characterized specific phenotypic abnormalities (Flynn et al., 1993; Mulley et al., 1995; Gecz et al., 1996). Repeated expansion of more than 200 copies of the CCG triplet of the 5′ untranslated region at the fragile X site is the main genetic cause of FRAXE syndrome. However, the literature reports other intragenic variants of the *AFF2* gene associated with clinical features that do not involve FRAXE syndrome. Strikingly, some of these individuals exhibit dysmorphic features that resemble those often seen in patients with CdLS, such as synophrys, anteverted nares, prominent nasal tip, cleft palate, microcephaly, clinodactyly, or abnormality of the digestive system (Valentino et al., 2021; Sahoo et al., 2011; ClinVar: VCV000986210.2; DECIPHER: 273134 and 323383). In addition, other features beyond those typically expected in CdLS are also reported, such as macrosomia, tall stature, or macrocephaly (da Rocha et al., 2014; ClinVar: VCV000986210.2; DECIPHER: 323383). Therefore, all these features suggest that the underlying clinical features in patients carrying intragenic variants in the *AFF2* gene would not be explained by the clinical picture described for FRAXE syndrome. However, an exhaustive comparison has been proved to be difficult as the clinical description of these cases is often fragmentary. Based on these distinctions, it can be concluded that our case does not align with FRAXE syndrome but with the spectrum of CdLS.

Notably, there is a functional association between *AFF2* and the other CdLS genes, all involved in transcriptional regulation. Cohesin plays a crucial role in the recruitment of chromatin remodelers and transcription factors to promoters. It also interacts with the mediator and stabilizes its interaction with RNA polymerase II (Singh et al., 2023). Similarly, the AFF2 protein participates in transcriptional regulation, forming the superelongation complex like-2 (SEC like-2) along with other factors. This complex is known to regulate RNA polymerase II activity (Luo et al., 2012; Luo et al., 2012b), particularly by preventing its paused state (Eigenhuis et al., 2022). Therefore, the association of CdLS phenotype with a *AFF2* variant can be also explained at the molecular level.

Based on the comprehensive clinical and molecular findings of this study, the intragenic duplication in the *AFF2* gene appears to be responsible for the clinical phenotype observed in our affected patients. The phenotype, combined with the known functional role of AFF2, underscores its relevance within the CdLS spectrum. Future research should focus on analyzing larger cohorts of patients with *AFF2* variants in combination with detailed clinical phenotyping to establish definitive genotype-phenotype correlations. Furthermore, the findings from this study suggest that individuals exhibiting a CdLS spectrum clinical presentation but lacking a molecular diagnosis, should be considered for *AFF2* gene analysis.

## Data Availability

The original contributions presented in the study are publicly available. This data can be found here: Leiden Open Variation Database (LOVD), variant ID: 00456114; https://databases.lovd.nl/shared/individuals/00456114.
