# Supplementary material for: An intragenic duplication in the AFF2 gene associated with Cornelia de Lange syndrome phenotype
Source: Front Genet. 2024 Nov 1;15:1472543. doi: 10.3389/fgene.2024.1472543 (PMC11563810; doi:10.3389/fgene.2024.1472543)
Supplement: Supplementary file 2 [file Table1.DOCX]

Supplementary Material

**Supplementary Table 1.** **Algorithm for the clinical diagnosis of Cornelia de Lange syndrome (CdLS) established by the International CdLS Consensus Group.**

| Clinical CdLS score | Patient (II-1) | Mother (I-2) | Aunt (I-3) |
| --- | --- | --- | --- |
| **CARDINAL FEATURES (2 points each if present)** |  | | |
| Synophrys (HP:0000664) and/or thick eyebrows (HP:0000574) | x |  | x |
| Short nose (HP:0003196), concave nasal ridge (HP:0011120) and/or upturned nasal tip (HP:0000463) |  | x | x |
| Long (HP:0000343) and/or smooth philtrum (HP:0000319) | x | x | x |
| Thin upper lip vermilion (HP:0000219) and/or downturned corners of mouth (HP:0002714) | x | x | x |
| Hand oligodactyly (HP:0001180) and/or adactyly (HP:0009776) |  |  |  |
| Congenital diaphragmatic hernia (HP:0000776) |  |  |  |
| **SUGGESTIVE FEATURES (1 point each if present)** |  | | |
| Global developmental delay (HP:0001263) and/or intellectual disability (HP:0001249) | x |  | x |
| Prenatal growth retardation (<2 SD) (HP:0001511) | x |  |  |
| Postnatal growth retardation (<2 SD) (HP:0008897) | x |  | x |
| Microcephaly (prenatally and/or postnatally) (HP:0000252) | x |  | x |
| Small hands (HP:0200055) and/or feet (HP:0001773) | x |  | x |
| Short fifth finger (HP:0009237) | x |  | x |
| Hirsutism (HP:0001007) | x |  | x |

The International CdLS Consensus Group defined a diagnostic algorithm in which the most common signs of the disorder are categorized as cardinal features or suggestive features, assigning each a specific score. According to the final score of the individual, the individual can be classified as classic CdLS (≥11 points, of which at least 3 are cardinal), non-classic CdLS (9 or 10 points, of which at least 2 are cardinal), molecular testing for CdLS indicated (4–8 points, of which at least 1 is cardinal) or insufficient to indicate molecular testing for CdLS (<4 points).


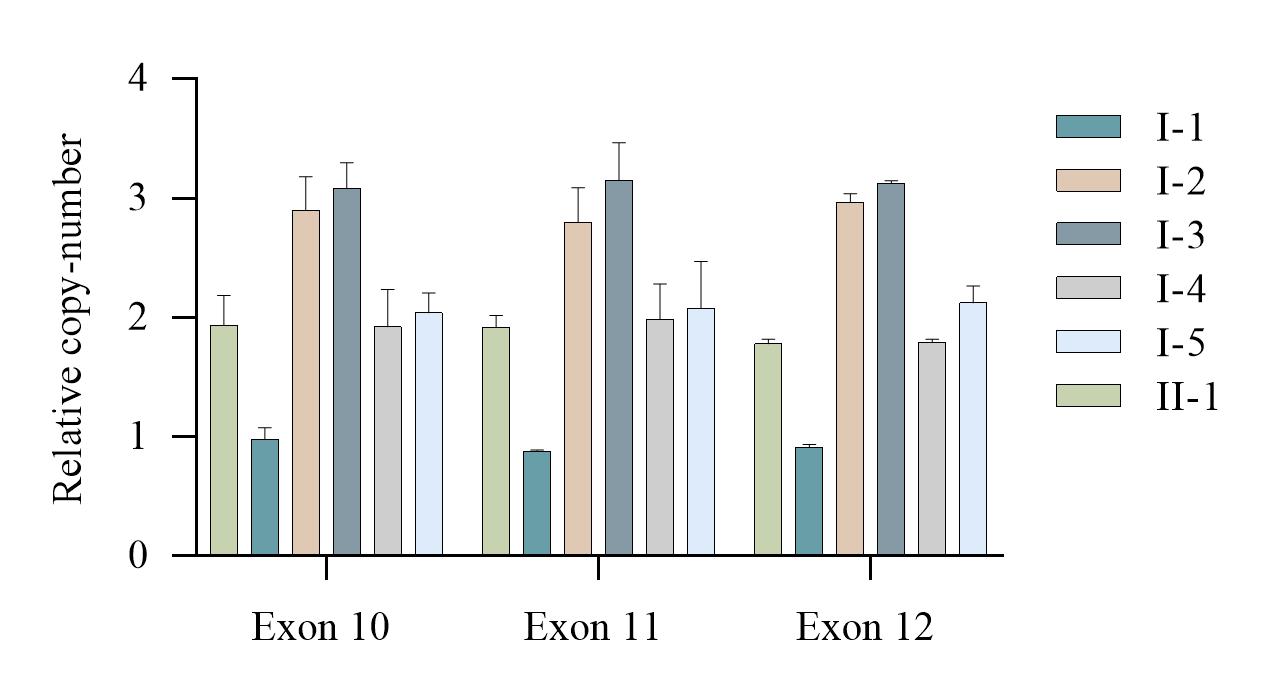


**Supplementary Figure 1.** Confirmation of familial segregation of the intragenic duplication in the *AFF2* gene. It was performed by qPCR on the patient (II-1), his father (I-1), mother (I-2), affected maternal aunt (I-3) and two unaffected maternal aunts (I-4 and I-5).


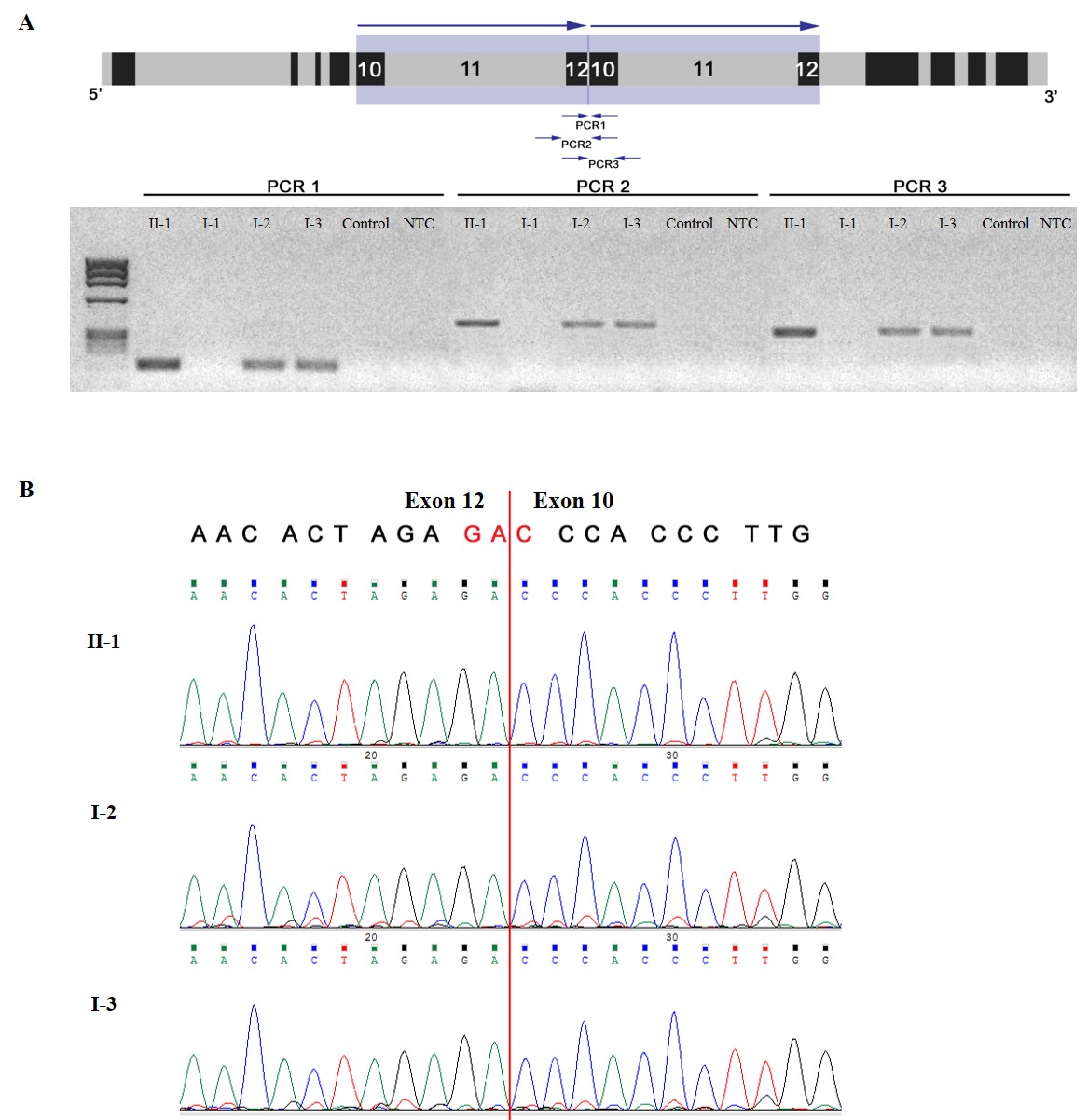


**Supplementary Figure 2.** Breakpoint identification. **(A)** Agarose gel of blood cDNA PCR products. Primers binding sites are shown (NTC: no template control; C: control). (**B)** Sanger chromatogram revealed that the duplication is arranged in a direct tandem orientation in the patient (II-1), his mother (I-2) and maternal aunt (I-3).


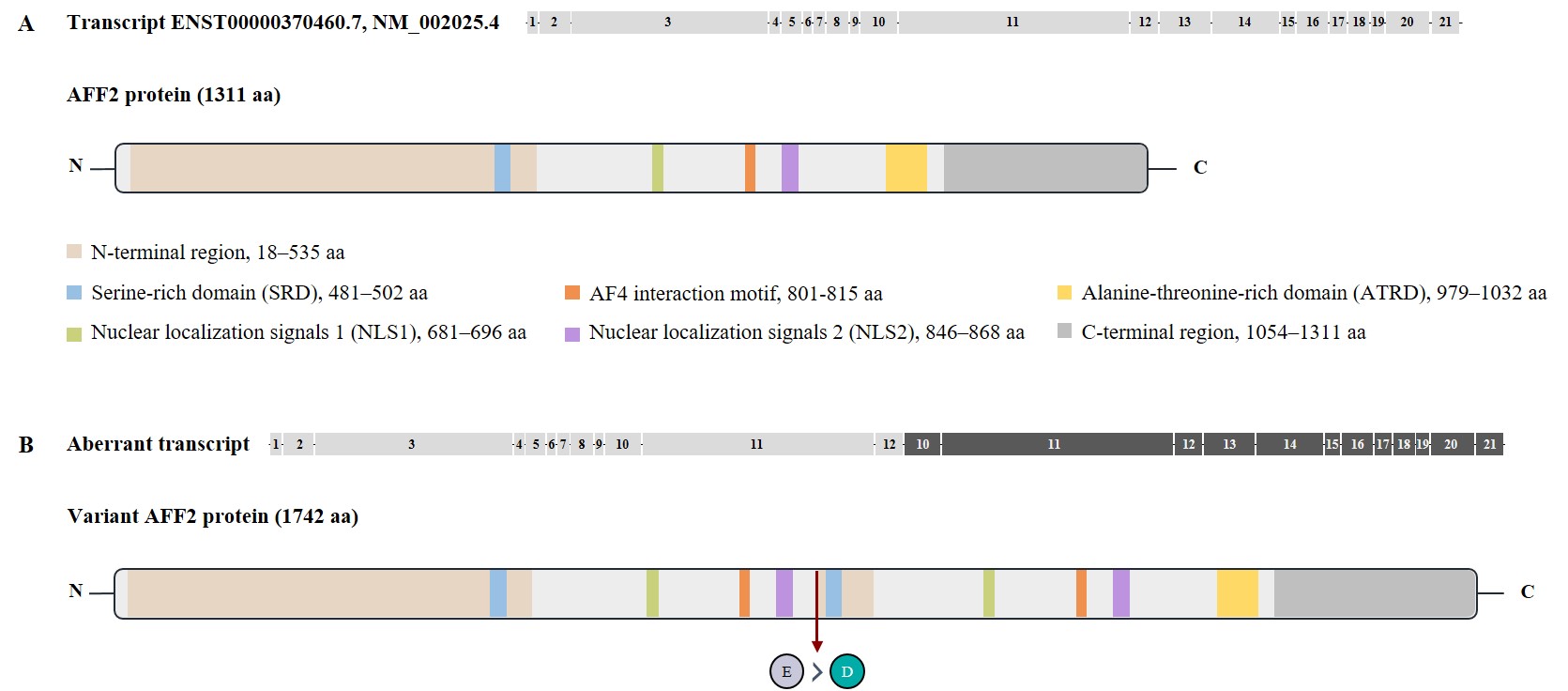


**Supplementary Figure 3.** Scheme of full-length AFF2 protein, indicating the NT domain (beige, 18-535 aa), the serine-rich domain (SRD, blue, 481-502 aa), the nuclear localization domain 1 (NLS1, green, 681-696 aa) and 2 (NLS2, purple, 846-868 aa), the AF4 interaction motif (orange, 801-815 aa), the alanine-threonine-rich domain (ATRD, yellow, 979-1032 aa) and the CT domain (grey, 1054-1311 aa). **(A)** The wildtype AFF2 protein (1311 aa). **(B)** The possible variant protein (1742 aa) in which a missense exchange of Glu897 to Asp897 was found.
